# Comorbidity screening in hidradenitis suppurativa: Evidence-based recommendations from the US and Canadian Hidradenitis Suppurativa Foundations

**DOI:** 10.1016/j.jaad.2021.01.059

**Published:** 2021-01-23

**Authors:** Amit Garg, Neeta Malviya, Andrew Strunk, Shari Wright, Afsaneh Alavi, Raed Alhusayen, Ali Alikhan, Steven D. Daveluy, Isabelle Delorme, Noah Goldfarb, Wayne Gulliver, Iltefat Hamzavi, Tarannum Jaleel, Alexa B. Kimball, Joslyn S. Kirby, Mark G. Kirchhof, Janice Lester, Hadar Lev-Tov, Michelle A. Lowes, Robert Micheletti, Lauren A. Orenstein, Vincent Piguet, Christopher Sayed, Jerry Tan, Haley B. Naik

**Affiliations:** a Department of Dermatology, Donald and Barbara Zucker School of Medicine at Hofstra/Northwell, New Hyde Park; b Division of Dermatology, Faculty of Medicine, Women College Hospital, University of Toronto, Toronto; c Department of Medicine and Sunnybrook Research Institute, University of Toronto, Toronto; d Department of Dermatology, Sutter Medical Foundation, Sacramento; e Department of Dermatology, Wayne State University School of Medicine, Detroit; f Dr Isabelle Delorme Inc, Dermatologue, Drummondville; g Departments of Internal Medicine and Dermatology, Minneapolis Veterans Affairs Health Care System, Minneapolis; h Faculty of Medicine, Memorial University of Newfoundland, St. John’s; i Multicultural Dermatology Center, Department of Dermatology, Henry Ford Hospital, Detroit; j Department of Dermatology, Duke University School of Medicine, Durham; k Harvard Medical Faculty Physicians at Beth Israel Deaconess Medical Center, Boston; l Penn State Milton S Hershey Medical Center, Hershey; m Division of Dermatology, Faculty of Medicine, University of Ottawa and The Ottawa Hospital, Ottawa; n Clinical Medical Library, Donald and Barbara Zucker School of Medicine at Hofstra/Northwell, Hempstead; o Dr. Phillip Frost Department of Dermatology and Cutaneous Surgery, University of Miami, Miller School of Medicine, Miami; p The Rockefeller University, New York; q Departments of Dermatology and Medicine, Perelman School of Medicine, University of Pennsylvania, Philadelphia; r Department of Dermatology, Emory University School of Medicine, Atlanta; s Women’s College Hospital, Toronto; t Department of Dermatology, University of North Carolina at Chapel Hill School of Medicine, Chapel Hill; u Department of Internal Medicine, University of Western Ontario, Windsor Campus; v Department of Dermatology, University of California, San Francisco

**Keywords:** acne, cardiovascular disease, comorbidity, Crohn’s disease, depression, dermatologist, diabetes mellitus, dissecting cellulitis of the scalp, down syndrome, dyslipidemia, generalized anxiety disorder, guidelines, herpes zoster, hidradenitis suppurativa, hypertension, inflammatory bowel disease, lymphoma, metabolic syndrome, North America, obesity, pilonidal disease, polycystic ovary syndrome, pyoderma gangrenosum, screening, sexual dysfunction, smoking, spondyloarthritis, substance use, suicide, systemic, ulcerative colitis

## Abstract

**Background:**

Hidradenitis suppurativa (HS) is associated with comorbidities that contribute to poor health, impaired life quality, and mortality risk.

**Objective:**

To provide evidence-based screening recommendations for comorbidities linked to HS.

**Methods:**

Systematic reviews were performed to summarize evidence on the prevalence and incidence of 30 comorbidities in patients with HS relative to the general population. The screening recommendation for each comorbidity was informed by the consistency and quality of existing studies, disease prevalence, and magnitude of association, as well as benefits, harms, and feasibility of screening. The level of evidence and strength of corresponding screening recommendation were graded by using the Strength of Recommendation Taxonomy (SORT) criteria.

**Results:**

Screening is recommended for the following comorbidities: acne, dissecting cellulitis of the scalp, pilonidal disease, pyoderma gangrenosum, depression, generalized anxiety disorder, suicide, smoking, substance use disorder, polycystic ovary syndrome, obesity, dyslipidemia, diabetes mellitus, metabolic syndrome, hypertension, cardiovascular disease, inflammatory bowel disease, spondyloarthritis, and sexual dysfunction. It is also recommended to screen patients with Down syndrome for HS. The decision to screen for specific comorbidities may vary with patient risk factors. The role of the dermatologist in screening varies according to comorbidity.

**Limitations:**

Screening recommendations represent one component of a comprehensive care strategy.

**Conclusions:**

Dermatologists should support screening efforts to identify comorbid conditions in HS.

As a chronic suppurating inflammatory disease, hidradenitis suppurativa (HS) exemplifies the potential link between integumentary and systemic disease. Indeed, HS is associated with higher overall comorbidity burden than exists for either healthy or psoriasis populations.^1^ This burden also influences mortality risk.^2^ The disproportionate prevalence of HS in women and Black patients ^3^ intensifies the need to identify comorbidities and mitigate their impact. Herein, the US and Canadian HS Foundations have provided evidence-based screening recommendations to support early recognition and treatment of comorbid conditions with the goal of improving long-term health outcomes for patients with HS.

## METHODS

The scope, content, synthesis of evidence, method of rating quality of evidence,^4^ and provisos are described in the Supplemental Materials (available via Mendeley at https://doi.org/10.17632/j52xjybbhg.1).

### Recommended role of the dermatologist in screening

Some screenings may be out of scope for dermatologists. We recommend that dermatologists perform examinations for comorbid conditions involving the skin and a simple review of systems for extracutaneous comorbidities. Individual screenings may be prioritized and distributed across visits as is feasible. Dermatologists may serve as advocates by directing primary physicians to potential comorbidities for which screening is recommended.

## RESULTS

The level of evidence and screening recommendations, including suggested methods and roles for the dermatologist, are summarized in Tables I and II.^5–13^

### Integumentary disease

#### Acne.

Like HS, acne is a disease of the pilosebaceous unit that affects young adults, has female predominance, and may be mediated by androgens. Acne and HS may also share common inflammatory pathways involving the follicular unit.

Higher prevalence of acne vulgaris/conglobata among patients with HS compared to control individuals was observed across 4 studies, with the prevalences ranging from 4.5% to 15.2%.^14–17^ The odds of having acne vulgaris/conglobata were 1.77 to 5.07 times greater in patients with HS.^14–17^ There were no large-scale studies addressing the prevalence of acne fulminans in patients with HS.

#### Dissecting cellulitis of the scalp.

Dissecting cellulitis of the scalp (DCS) and HS are suppurative conditions involving the follicular unit characterized by perifollicular inflammatory nodules and interconnected draining tunnels eventuating in scarring. Some consider DCS to represent HS of the scalp.

In one study of more than 5000 patients with HS, DCS prevalence was 9.2%, compared to 0.7% in matched control individuals.^17^ The risk of DCS in patients with HS was 13.38 (95% confidence interval [CI], 9.60–18.64) times greater.^17^

#### Pilonidal disease.

Pilonidal disease (PD) and HS are chronic inflammatory diseases of follicular occlusion characterized by perifollicular inflammatory nodules and draining tunnels.

Across 2 large-scale studies and 1 small retrospective cohort study, PD prevalences among patients with HS ranged from 1.4% to 2.3%, compared with 0.1% to 0.3% in control individuals.^15–17^ The prevalence of PD may be greater among patients with severe HS.^17^ Adjusted odds of PD among patients with HS ranged from 4.97 to 5.61 times that of control individuals.^15–17^

#### Pyoderma gangrenosum.

HS and pyoderma gangrenosum (PG) share clinical features, including similar demographic composition, presence of suppurating plaques and ulcers, neutrophil rich inflammation, response to tumor necrosis factor (TNF) inhibitors, and coexistence as part of autoinflammatory syndromes.

The prevalence of PG among patients with HS ranges from 0.2% to 0.4%.^16,18^ Prevalence is higher (3.7%) among patients with HS having Crohn’s disease (CD).^18^ In a study of 65,000 patients with HS, the adjusted odds of having PG was up to 20 times greater among patients with HS (0.18%) compared to control individuals (0.01%).^18^ The association was independent of CD status.^18^

#### Herpes zoster.

Local and systemic inflammation and exposures to immunomodulating therapies may increase reactivation risk. Introduction of a highly effective herpes zoster (HZ) vaccine has also increased the relevance of identifying individuals at elevated risk.

In a retrospective study, hospitalized patients with HS had 1.65 (95% CI, 1.13–2.39) times the odds of HZ compared to hospitalized control individuals, although absolute prevalence was low in both groups (0.143% vs 0.145%).^19^ In another retrospective cohort study of 30,000 patients with HS, those not receiving immunosuppression had an incidence of 0.4%.^20^ Patients with exposure to immunosuppression had a crude HZ incidence of 1.3%, although this burden still approximated that observed in the general US population (1.0%).^20^ The adjusted odds ratio (aOR) of developing HZ among patients with HS was similar regardless of immunosuppression status, suggesting that immunosuppression does not modify the relationship between HS and HZ.^20^

### Mood disorder and suicidality

The pathogenesis of HS and major depression may be linked through increased expression of proinflammatory cytokines, including interleukin (IL) 6 and TNF, which are observed at higher levels in both conditions.^21^ Additionally, HS has substantial impact on health-related quality of life,^22^ which likely contributes to risk of depression, anxiety, and suicidality.

#### Depression.

The prevalence of depression in HS populations is as high as 26%.^15^ The adjusted odds of depression among patients with HS ranges from 1.3 to 4.8 times that of control populations.^15,23–25^ The prevalence of depression may be greater among patients with a higher Hurley stage.^26^

#### Generalized anxiety disorder.

In a meta-analysis, the prevalence of generalized anxiety disorder among patients with HS was approximately 5%.^27^ In a prospective community-based study, patients with HS had 1.7 times the odds of anxiety compared to control individuals.^28^ Larger retrospective clinical and administrative database studies have also described higher prevalences and likelihoods of anxiety among patients with HS compared to control individuals.^17,24^

#### Completed suicide.

Evidence from European national registries indicates higher incidence of completed suicide among patients with HS. In a Finnish study, 4.4% of deaths in patients with HS were caused by suicide compared to 1.8% of deaths in control individuals, and risk of suicide was 2.8 (95% CI, 1.7–4.5) times higher in patients with HS.^29^ In Denmark, patients with HS were observed to have a crude incidence of completed suicide of 0.3 per 1000 person-years, with an increased adjusted risk of 2.4 (95% CI, 1.1–5.5) times that of control individuals. ^28^ The risk of suicide may be higher among women with HS.^29^

### Substance use

#### Tobacco smoking.

Nicotinic acetylcholine receptors are found on cells putatively implicated in the pathogenesis of HS. Nicotine has been shown to induce infundibular epithelial hyperplasia and hyperkeratosis, alter the cutaneous microbiome, stimulate release of TNF by keratinocytes and T-helper 17 cells, disturb polymorphic neutrophil granulocyte chemotaxis, and immunomodulate macrophage function.

Five cross-sectional studies from Europe,^23,30,31^ South America,^32^ and Turkey^33^ observed significantly higher frequencies of self-reported smoking among patients with HS compared to control individuals, with prevalences in HS ranging from 17.9% to 88.9%. Two large retrospective studies in North America^34^ and Israel^35^ and a single-center case-control study^36^ also found a significant association between smoking and HS by using electronic records. In one of these, a 90% increase in risk (aOR, 1.9; 95% CI, 1.8–2.0) of new HS diagnosis was observed among smokers compared to nonsmokers, suggesting that smoking could be a risk factor for HS.^34^ Evidence supporting smoking cessation as a means to improve the disease course is limited.

#### Substance use disorder.

Patients with HS experience chronic pain and have physical, emotional, and psychological disease impact which may increase risk for substance use disorder (SUD).

In a cross-sectional study in 32,000 patients with HS, the prevalence of SUD was 4.0%, compared to 2.0% in control individuals. The most common forms of SUD were alcohol and opioid use. Patients with HS had 1.50 times the adjusted odds of SUD (aOR, 1.50; 95% CI, 1.42–1.59).^37^ In a retrospective cohort study involving 20,000 patients with HS, the 1-year incidence of chronic opioid use among opioid-naive patients with HS was 0.3%, double that of control individuals. In adjusted analysis, patients with HS had 1.5 times greater risk (aOR, 1.5; 95% CI, 1.2–2.0) of developing chronic opioid use.^38^ In a single-center case-control study of 1800 patients with HS, diagnoses of alcohol dependence (4.2% vs 0.5%) and drug dependence (6.5% vs 0.5%) were more common in patients with HS compared to control individuals, although adjustment for comorbidities reversed or attenuated these associations.^36^

### Endocrine disease

#### Polycystic ovary syndrome.

Both HS and polycystic ovary syndrome (PCOS) affect similar demographics, may be complicated by obesity and metabolic syndrome, and respond to antiandrogen agents.

In a cross-sectional analysis involving 23,000 women with HS, the prevalence of PCOS was 9.0%, compared to 2.9% in control individuals. Women with HS had twice the adjusted odds of having PCOS (aOR, 2.14; 95% CI, 2.04–2.24).^39^ In other retrospective database studies evaluating various comorbid outcomes, the prevalences of PCOS among women with HS ranged from 0.8% to 4%. Across these studies, women with HS had 1.2 to 13.4 times the odds of having PCOS compared to control individuals.^15,17,40^

### Metabolic disease

The biological link between HS and metabolic syndrome (MetS) may be explained by the downstream systemic effects of adipose tissue secretion of proinflammatory mediators in obesity along with insulin resistance in the setting of chronic inflammation. Impaired Notch signaling in HS may result in elevations of TNF and subsequent impairment of insulin signaling.^41^ Hyperinsulinemia in the setting of obesity may also lead to overactivation of mTORC1 signaling via insulin-like growth factor, resulting in insulin resistance.

#### Obesity.

A higher prevalence of obesity among patients with HS compared to control individuals was observed across 7 studies, with prevalences ranging from 5.9% to 73.1%.^14,23,29,32,35,36,42^ In a meta-analysis of 8 studies, the adjusted pooled odds of obesity among patients with HS was 3.5 (95% CI, 2.2–5.4) times that of control individuals.^43^ Evidence supporting weight loss as a means of improving the disease course is limited.

#### Dyslipidemia.

A higher prevalence of dyslipidemia among patients with HS compared to control individuals was observed across 4 studies, with prevalences ranging from 3.3% to 45.3%.^15–17,36^ Adjusted odds of dyslipidemia among patients with HS ranged from 1.4 to 4.1 times that of control individuals.^15–17^ In a meta-analysis of 9 studies comprising 6174 patients with HS, the odds of hypertriglyceridemia and low high-density lipoprotein levels among patients with HS were 1.7 (95% CI, 1.1–2.5) and 2.5 (95% CI, 1.5–4.2) times that of control individuals, respectively.^43^

#### Diabetes mellitus.

A higher prevalence of diabetes mellitus (DM) among patients with HS compared to control individuals was observed across 7 studies, with prevalences ranging from 7.1% to 24.8%.^15,17,35,36,44–46^ In 2 meta-analyses of 12 and 7 studies, the pooled odds of DM among patients with HS were 2.17 (95% CI, 1.9–2.6)^40^ and 2.8 (95% CI, 1.8–4.3)^47^ times that of control individuals, respectively.

#### MetS.

Higher prevalences of MetS among patients with HS compared to control individuals were observed across 5 studies, with prevalences ranging from 10.4% to 50.6%.^35,42,46,48,49^ The pooled adjusted odds of MetS among patients with HS ranged from 1.8 to 2.2 times that of control individuals.^40,43,50^

### Cardiovascular disease

#### Hypertension.

Obesity and tobacco use are behavioral risk factors for hypertension that are associated with HS. Patients with HS had significantly higher prevalence of hypertension in 6 of 8 total studies addressing this relationship, with aORs ranging from 1.2 to 2.1.^15–17,35,36,42,46,51^ In these studies, the prevalence of hypertension among patients with HS ranged from 7.8% to 56.3%. In 1 of these studies, the risk of hypertension was lower in patients with HS (OR, 0.7; 95% CI 0.6–0.7] than control individuals, although data from this study were limited to single inpatient admissions and may not have included past comorbidity information.^51^

#### Major adverse cardiovascular events.

HS is associated with cardiovascular risk factors including obesity, smoking, DM, and dyslipidemia. Circulating TNF and IL-6 are elevated in HS and known to drive endothelial dysfunction, atherosclerosis, and thrombosis.^52,53^ As such, chronic systemic inflammation may support a biological link between HS and cardiovascular disease.

The composite of myocardial infarction, cerebrovascular accident, and cardiovascular death, also referred to as *major adverse cardiovascular events* (*MACE*), are associated with HS. In a retrospective population-based cohort study, incidence rates of MACE among patients with HS and control individuals were 40 and 19 per 10,000 person-years, respectively.^54^ The adjusted incident risk of MACE among patients with HS was 1.5 (95% CI, 1.27–1.86) times that of control individuals, and the adjusted risk of cardiovascular death among patients with HS was 1.6 (95% CI, 1.2–2.1) times that of patients with psoriasis.^54^ In a single-center study, patients with HS had greater mean carotid intima-media thickness than control individuals. Patients with HS had carotid plaques more frequently, with a 3 times greater risk of carotid plaques (aOR, 3.0; 95% CI, 1.26–7.13].^55^ In a prospective observational study, subclinical atherosclerosis was present in 30.6% of patients with HS compared to 16.1% of control individuals.^56^ The odds in patients with HS older than 40 years were 3.0 times greater than in control individuals (95% CI, 1.0–9.5).^56^ Resting heart rate, which is linked to all-cause and cardiovascular death, was associated with severe HS (adjusted mean difference vs control individuals, 2.9 beats/min; 95% CI, 0.7–5.1; *P* = .01).^57^

### Gastrointestinal disease

#### Inflammatory bowel disease.

Ulcerative colitis (UC), CD, and HS are all chronic, recurrent, inflammatory disorders of the epithelia with the inhabitation of commensal flora. HS and CD are further characterized by suppuration and granulomatous inflammation, which may eventuate in fistula and sinus tract formation. Onset typically occurs in young adulthood. Spondyloarthropathy is also linked to all 3. These diseases may also share overlapping cytokine signatures with response to corresponding targeted therapies.

The prevalence of CD among 50,000 patients with HS was 2%, compared to 0.6% among non–HS patients.^58^ The risk of CD among patients with HS was 3 times greater (aOR, 3.05; 95% CI, 2.87–3.25).^58^ In 3 other studies, prevalences of CD among patients with HS ranged from 0.2% to 0.8%.^16,59–61^ Among patients with HS, the adjusted odds of having CD were 1.2 to 2.0 times that of control individuals.^16,59–61^ The prevalences of UC among patients with HS ranged from 0.3% to 1.3%.^16,59,60^ In adjusted analyses from 3 population-based studies, patients with HS had 1.3 to 1.8 times the odds of UC compared to control individuals.^16,59,60^

### Musculoskeletal disease

#### Spondyloarthritis.

Spondyloarthritis and HS share key inflammatory mediators including TNF, IL-1, and IL-17, which may suggest a biological link between these conditions. In 2 studies, the prevalences of spondyloarthritis among patients with HS were 0.3% and 28% compared with 0.2% and 2.6%, respectively, among control individuals.^16,62^ In 1 study of 1700 patients with HS, the prevalence of spondyloarthropathies was 52.5%, compared to 3% among control individuals.^36^ The adjusted odds of spondyloarthritis among patients with HS was 1.5–9.4 times greater.^16,62^

### Urogynecologic disease

#### Sexual dysfunction.

Because HS involves the inframammary folds, groin, genitals, and buttocks, patients with HS may be at risk for sexual dysfunction (SD), including abnormal sexual function, impairment, and distress.

Three prospective cross-sectional survey studies consistently showed significant differences in sexual health between patients with HS and control individuals using a variety of validated sexual health measures.^63–65^ One study observed that both male and female patients with HS reported significantly lower sexual function compared to control individuals, as measured by the International Index of Erectile Function and Female Sexual Function Index.^64^ In a study involving more than 40,000 patients with HS, the 5-year cumulative incidence of SD was 1.7%, compared to 1.5% in control individuals.^66^ The risk of SD among patients with HS was approximately 1.4 (95% CI, 1.3–1.5) times that of control individuals, regardless of patient sex.^66^

### Genetic disorders

#### Down syndrome.

The expression of amyloid precursor protein is increased in DS. In the epidermis, amyloid precursor protein plays a role in stimulating keratinocyte adhesion, migration, and proliferation. In this way, patients with Down syndrome (DS) may be prone to keratinocyte hyperproliferation and follicular plugging.

In a cross-sectional study of nearly 12,000 patients with DS, the prevalence of HS was 2.1%, compared to 0.3% in control individuals.^67^ Patients with DS had 5 times the prevalence of HS (aOR, 5.24; 95% CI, 4.62–5.94) compared to patients without DS.^67^ More than 80% of patients with DS had their HS diagnosis by age 29 years. In a single-center study, the prevalence of HS among patients with DS was 24%, compared to 1.2% among control individuals.^68^

### Comorbid conditions for which the systematic review did not support screening

Conditions for which insufficient evidence exists to make recommendations on screening^16,17,69–72^ or for which screening was not recommended^15–17,45,73–75^ are discussed in the Supplemental Materials.

### Gaps in research for comorbidities

Several remaining knowledge gaps in our understanding of HS comorbidities are discussed in the Supplemental Materials.

## SUMMARY

A number of comorbid conditions affect patients with HS and contribute to poor health and impaired quality of life, beyond the significant impact of the disease itself. These screening guidelines are intended to support health advocacy efforts along with comprehensive care strategies for patients with HS.

## Figures and Tables

**Table I.** Level of evidence and strength of comorbidity screening recommendations in HS

Comorbidity in HS	Level of evidence	Strength of recommendation	Is screening recommended?*

Acne vulgaris/conglobata	II	B	Yes
Dissecting cellulitis of scalp	II	B	Yes
Pilonidal cyst	II	B	Yes
Pyoderma gangrenosum	II	B	Yes, for patients with ulcerations, regardless of inflammatory bowel disease status
Depression	II	B	Yes
Anxiety	II	B	Yes
Suicidality	II	B	Yes, for patients who have known psychiatric disease, including substance use, or those who exhibit signs of psychological distress
Tobacco	II	B	Yes
Substance misuse	II	B	Yes, for patients with chronic pain, depression, or anxiety
Polycystic ovary syndrome	II	B	Yes
Obesity	II	B	Yes
Dyslipidemia	II	B	Yes
Diabetes mellitus	II	B	Yes
Metabolic syndrome	II	B	Yes
Hypertension	II	B	Yes
Cardiovascular disease	II	B	Yes
Inflammatory bowel disease	II	B	Yes
Spondyloarthritis	II	B	Yes
Sexual dysfunction	II	B	Yes
Down syndrome	II	B	Yes, screen patients with trisomy 21 for HS
Thyroid disease	–	–	Insufficient evidence
Nonalcoholic fatty liver disease	–	–	Insufficient evidence
Obstructive sleep apnea	–	–	Insufficient evidence
Renal disease	–	–	Insufficient evidence
Sleep disturbances	–	–	Insufficient evidence
Alzheimer disease	II	B	No
Herpes zoster	II	B	No
Lymphomas	II	B	No
Psoriasis vulgaris	II	B	No

*HS*, Hidradenitis suppurativa.

* A recommendation for or against screening is based on the (1) findings and overall level of evidence from the systematic review, including the absolute prevalence of the condition and magnitude of association with HS; (2) potential benefits, harms, costs, and feasibility of screening; and (3) latest population-based screening recommendations in the general population, if available.

**Table II.** Suggested screening methods and frequency for comorbid conditions in HS

Comorbidity in HS	Screening method	Suggested frequency

Screening performed by dermatologist using physical examination; manage if positive screening result		
Acne vulgaris/conglobata	Physical examination of face and trunk	Annual
Dissecting cellulitis of scalp	Physical examination of scalp	Annual
Pilonidal disease	Physical examination of sacral region	Annual
Pyoderma gangrenosum	Physical examination of ulcerations	With presence of cutaneous ulcerations
Down syndrome	Physical examination of patients with Down syndrome for findings suggestive of HS	Annual
Screening performed by dermatologist using screening question; refer for management with positive screening result		
Tobacco smoking	Screening question: “In the past year, how often have you used tobacco products?”^5^	Annual
Inflammatory bowel disease	Screening question: “Have you had abdominal pain at least 3 times a week for at least 4 weeks, bloody stools, diarrhea (more than 3 bowel movements daily) for 7 consecutive days, or been awoken from sleep because of abdominal pain or diarrhea?”^6^	Annual
Spondyloarthritis	Screening question: “Do you have joint pain or stiffness that is worse first thing in the morning or after a period of inactivity and gets better as the day goes on?”	Annual
Sexual dysfunction	Screening question: “Have you been sexually active in the past 6 months? Do you or your partner have any sexual difficulties, such as your interest level or intercourse-related pain?”	Annual
Screenings referred to primary care or other specialty for screening and management		
Obesity	Measurement of height and weight with calculation of body mass index^7^	Determined by physician to whom patient is referred
Depression	PHQ-2 and PHQ-9^8^	Determined by physician to whom patient is referred
Generalized anxiety disorder	GAD-7^9^	Determined by physician to whom patient is referred
Suicidality	Item 9 of the PHQ-9 assessing thoughts of self-harm^10^	Determined by physician to whom patient is referred
Substance use disorder	Alcohol: AUDIT-C questionnaire^11^ Opioid Risk Tool^12^	Determined by physician to whom patient is referred
Polycystic ovary syndrome	Rotterdam criteria with ≥2 of the following: oligo/anovulation, clinical or biochemical hyperandrogenism, and polycystic ovaries on transvaginal ultrasonography.^13^	Determined by physician to whom patient is referred
Hypertension	Blood pressure measurement^7^	Determined by physician to whom patient is referred
Dyslipidemia	Fasting lipid panel^7^	Determined by physician to whom patient is referred
Diabetes mellitus	Glycated hemoglobin or fasting blood glucose^7^	Determined by physician to whom patient is referred
Metabolic syndrome	Abnormality in ≥3 of the following: blood pressure measurement, fasting triglyceride, fasting HDL, fasting blood glucose, waist circumference measurement^7^	Determined by physician to whom patient is referred
Cardiovascular disease	Anthropometric measurements, waist circumference measurement, blood pressure measurement, fasting lipid panel, fasting blood glucose, assessment of tobacco use, physical activity and diet^7^	Determined by physician to whom patient is referred

*AUDIT*, Alcohol Use Disorders Identification Test; *GAD*, generalized anxiety disorder; *HDL*, high-density lipoproteins; *HS*, hidradenitis suppurativa; *PHQ*, Patient Health Questionnaire.

## CAPSULE SUMMARY

- Hidradenitis suppurativa (HS) is associated with a high comorbidity burden.
- Dermatologists have a pivotal role in screening for some comorbidities in HS while also serving as advocates for patients with HS when referring for interdisciplinary screening measures.

## Abbreviations used:

aOR: adjusted odds ratio
CD: Crohn’s disease
CI: confidence interval
DCS: dissecting cellulitis of the scalp
DM: diabetes mellitus
DS: Down syndrome
HS: hidradenitis suppurativa
HZ: herpes zoster
IL: interleukin
MACE: major adverse cardiovascular events
MetS: metabolic syndrome
PCOS: polycystic ovary syndrome
PD: pilonidal disease
PG: pyoderma gangrenosum
SD: sexual dysfunction
SUD: substance use disorder
TNF: tumor necrosis factor
UC: ulcerative colitis
